# Review of Some of the Surgical Cases Which Have Lately Occurred in the Royal Infirmary of Edinburgh. A Clinical Lecture, Delivered to the Students of Surgery in That Institution, on Thursday, 26th February, 1829

**Published:** 1829-09

**Authors:** George Ballingall

**Affiliations:** Fellow of the Royal College of Surgeons, Surgeon Extraordinary to the King, Regius Professor of Military Surgery in the University of Edinburgh, and One of the Surgeons to the Royal Infirmary.


### DR. BALLINGALL'S CLINICAL LECTURE.

*Review of some of the Surgical Cases which have lately occurred in the* ROYAL INFIRMARY *of* EDINBURGH. *A Clinical Lecture, delivered to the Students of Surgery in that Institution, on Thursday,* 26*th February,* 1829, *by* GEORGE BALLINGALL, M.D. F.R.S.E.; Fellow of the Royal College of Surgeons, Surgeon Extraordinary to the King, Regius Professor of Military Surgery in the University of Edinburgh, and one of the Surgeons to the Royal Infirmary.

(Concluded from p. 103.)

THE next case to which I would solicit your attention is one altogether of a different nature, and which too seldom occurs so opportunely as to afford the students of public hospitals an opportunity of seeing the operation required in it. On the 4th of January, Elizabeth Thomson, æt. forty, was admitted with strangulated inguinal hernia, and the following detail of circumstances recorded in the journal:

"Has had a reducible inguinal hernia for nine or ten years, but within these last ten days it has become strangulated. Nausea and vomiting of stercoraceous matter has supervened. Much pain and debility is complained of.

"The operation was performed by Dr. Campbell, the stricture divided, and the gut reduced; to which was attached two or three diverticulæ of the intestine.

"5th.—Was bled to about fourteen ounces. One copious stool from injection; slept well; pulse 100, rather sharp; abdomen not tender; complains of slight nausea and soreness of the wound. Had castor oil this morning. Tongue moist.

"6th.—Pulse less sharp; tongue moist; slept well from anodyne draught; copious stool from injection last night; feels much easier this morning, and makes no complaint of pain."

From this period her cure went on progressively, and she was dismissed on the 26th of January.

The appearance of the diverticulæ or appendices to the intestine, which were seen at the time of the operation, constitutes one of those unforeseen circumstances which we are constantly meeting with in operations for strangulated hernia, and for which you should never be unprepared. But one of the most remarkable circumstances in this case was, that, notwithstanding the long continuance of the symptoms of strangulation, the tumor itself was by no means tense or inflamed: on the contrary, it felt rather flaccid, and not particularly tender to the touch. Dr.

Campbell, however, very properly determined upon an immediate operation, seeing that the woman's bowels had been obstructed for five days, and that he could make no impression on the tumor by the taxis. The patient was upon the operating table in less than half an hour after her admission into the house, and the beneficial effects of this promptitude you had an opportunity of witnessing. The functions of her bowels were speedily restored; the wound healed kindly, and in three weeks she left the house cured, adding another to the large proportion of successful results which we have experienced here from this operation within the last few years.

On the 11th of this month, a case of disease presented itself in another female, which has been of very rare occurrence during my connexion with the house: I allude to that of Betsy Dodds, æt. forty-one, who was admitted with scirrhus of the os uteri, and of whose situation the following particulars are detailed :

"States that about ten weeks ago she had stoppage of the menses, which was accompanied with severe pains shooting up the back from the situation of the uterus, also with bearing-down pains. These were always worse during the night. At the commencement of this attack, her health was considerably impaired. These lancinating pains still continue in the lower part of the abdomen, but more especially in the back. She now menstruates pretty regularly. She is married, and has eight of a family. She had the last child about four years ago. Has had no miscarriages."

On the 12th, Mr. Liston removed the os uteri by excision, and the part amputated was shown to you at a subsequent lecture. "There was oozing of blood to the extent of nearly a pound after the operation; she had also faintness and vomiting, with considerable pain in the uterus. The bleeding was stopped by the application of cloths dipped in cold water to the pubis; and she had an anodyne draught at bedtime, which was rejected by vomiting."

For some days after this, she had some quickness of pulse, thirst, and other febrile symptoms, with pain in the back. The sore was examined with the speculum two days ago, and found to have a healthy appearance.

In the few remarks which I offered you upon this case, I referred to the recent papers on the partial or total excision of the womb in various periodical works, and noticed particularly the statements of Lisfranc, which abundantly prove the safety of such operations, whatever may be thought of their general expediency; but, as I can afford you no further information on this subject from my own experience, I would refer you again to the valuable observations of Hesse upon excision of the womb, where you will find numerous details of this operation, as performed by Osiander and other eminent practitioners abroad: you will also do well to consult the writings of Lisfranc, of Paris, and of Blundell and others in this country.

Among several other cases of lithotomy, you had an opportunity of seeing a very large stone removed, by Mr. Liston, from the bladder of Janet Alexander, æt. thirty-eight, who was admitted on the 13th of November, and whose case is thus detailed in the journal:

"Has been troubled for the last four years with frequent desire to pass urine, which contains a good deal of sediment. Has most pain when bladder is empty. At commencement her urine was bloody. Has at times violent pain in her back. Has occasionally passed portions of gravel. On sounding her, a stone of pretty large size was found. Belly costive; health rather declining; catamenia regular. Had her last child two and a half years ago.

"16th.—Mr. Liston removed the stone today, which was found of a large size, (so much so, that he required for its extraction to make an incision upwards and outwards on both sides.) A tube was introduced. Has passed a quiet night; slept much; skin moist; urine flows freely through the tube.

"17th.—Passed a quiet night; little pain; urine flows freely through the tube; belly not open —Ol. Ricini ℥i.

"Did not sleep well; complains of some uneasiness about abdomen. Had an enema, by which her bowels were freely evacuated, since which she is easier. The tube was removed yesterday; urine flows in good quantity; pulse 100, skin moist, tongue moist.

"24th.—Continues to do well; sleeps pretty well; belly open; water is not retained yet; wound looks well; skin cool, pulse ninety-six; appetite improving.—Steak ℥iv. Vin. Hispan. ℥iv. daily.

"28th.—Is doing well, but is unable to retain her urine."

On the 3d of December she was dismissed, but with some incontinence of urine.

The operation for stone, you are all aware, is much less frequently required in the female than in the male subject, and of late we have had a number of cases recorded in which stones of a very large size have been removed by dilatation of the female urethra, without the use of cutting instruments. Many of these cases have been detailed apparently for the purpose of showing that the use of the knife is here altogether superfluous; but, from what I have seen of the practice of lithotomy in the female upon the present and upon two former occasions, I cannot believe that the sufferings of the patient, either present or remote, are at all to be compared to those occasioned by the forcible dilatation of the urethra. In the case now under consideration, the removal of the stone in its entire state by the dilatation of the urethra would, I believe, have been altogether impossible.

In the very extraordinary case of Andrew Leechman, æt. seventy, Mr. Liston gave you an opportunity of seeing the employment of an apparatus lately much used in France for the purpose of breaking down stones in the bladder. As this case is intended for publication I shall offer no comments upon it, but shall merely transcribe the following particulars from the case-book:*

"States that he has laboured under the following symptoms for five months: pain at the point of the penis; when making water in a full stream, it sometimes stops suddenly; has frequent desire to pass his urine, which, after any great exertion, is mixed with blood. There is a considerable deposit of mucus when urine is allowed to stand. Passed a small stone on Saturday.

"On introducing a sound, a stone is distinctly felt; prostate gland healthy; no pain in back; has always enjoyed good health; says that two years ago he made his water frequently, but without pain.—Descend. in baln."

On the 13th of November, three days after his admission, "Civiale's instrument was introduced yesterday by Mr. Liston; but, from the irritable state of the bladder, (though a solution of opium had been previously injected,) he was obliged to desist, without grasping the stone completely. Several small fragments, however, came away within the fangs of the instrument, and during the course of the night several pieces of stone have been passed. There was no bloody urine after operation, nor has he passed a worse night, but slept better than for some time past.

"15th.—Passed a barleycorn this morning, encrusted with calcareous matter; urine much improved.

"16th.—A small piece of wood was passed, with the same appearance as the barleycorn. Complains of pain in the scrotum.

"18th.—A small abscess which had formed in the scrotum was opened."

* London Medical and Physical Journal, May 1829, p. 412.—Ed.

On the 25th the instrument was again introduced, the stone was fairly laid hold of, but was so soft that it was crushed by the instrument, in withdrawing which several fragments of seeds were found adhering. Subsequent to this he passed several fragments of stone, having entire barleycorns for their nuclei. He now confessed that, while reaping last harvest, he had introduced a number of barleycorns into his urethra, but would not say for what purpose.

On the 19th December James M'Donald, a delicate looking boy of about ten years of age, was sent over from Perth to undergo an operation for the stone, and was admitted under my care with the following symptoms: "Complains of great pain in voiding his urine, more particularly towards the end of, and immediately after the evacuation, and there is a considerable quantity of mucus and blood occasionally mixed with his urine. On introducing a sound, a calculus can be distinctly felt. The disease is of three years' standing, and the symptoms have been gradually becoming more severe." Two days after this he was placed upon the operation-table; but, after a very strict and rather protracted examination, no stone could be felt with the staff, either by myself or any of my colleagues, and I was reluctantly compelled to send the patient to bed.

He was sounded in the consultation-room a few days afterwards, but without our being able to feel the stone; and, after each of these examinations, he passed large quantities of calculous matter, leading me to conjecture that, although the sound could not be distinctly felt nor heard to strike the stone, that it had detached this gritty matter from its surface, or had perhaps broken it completely down.

On the 9th of January he was sounded again, first with a steel catheter and afterwards with a small-sized sound, with which latter instrument the stone was distinctly struck by Mr. Liston, and afterwards by myself.

The little patient was brought into the theatre on the following day, and the operation performed with a grooved staff and a common scalpel. It was very gratifying, after the circumstances detailed in the history of this boy's case, to find that I was ultimately enabled to extract the stone with as much ease and expedition as I could ever desire. The patient recovered without a single bad symptom, and in three weeks was sent home cured.

The stone extracted from M'Donald is about the size of a large almond, and consists apparently of uric acid. It was, I am persuaded, on his admission, encrusted with an adventitious layer of the mixed phosphates. This I am induced to believe from the very large quantity of calculous matter passed after each examination, amounting, I am assured, to more than a dessertspoonful in all; and also from the different and more obtuse sound occasioned in striking the stone at the first examination, compared with the distinct stroke which was heard by all the by-standers at the time of the operation.

In reflecting upon the cause of our being unable to feel the stone upon two occasions, I am inclined to think that something was attributable to using too large a sound, which, being grasped by the urethra and sphincter of the bladder, did not move freely within it; and although it was subsequently changed for a smaller instrument, yet the bladder, having been previously irritated by the presence of the larger one, was probably excited to a spasmodic action, which impeded the discovery of the stone.

In my comments upon this case, I noticed various circumstances which occasionally render the detection of stones difficult. I mentioned a case of my own, where, although the stone was of a very large size, it was very indistinctly perceived by the sound, and we were only satisfied of its existence by feeling it through the abdominal parietes. I stated to you also the outlines of the case of a scientific gentleman in London, who was repeatedly sounded by some of the most eminent surgeons of the day, but not one of them could positively say that he felt the stone, until, in consequence of an accident, it was dislodged from the fundus of the bladder where it had hitherto lain, and was very readily detected by the sound. The same accident, however, which led to the satisfactory detection of a large calculus, led, in that instance, to the death of the patient within a few hours afterwards; and, upon dissection, a ruptured membranous rim was found surrounding the fundus of the bladder where the stone had been lodged.

It is not, however, in subjects of the age of M'Donald that occurrences of this kind are generally met with; and I stated, perhaps too strongly, that sacculated stones are seldom or never found in such young patients. A very remarkable case to the contrary, and one very creditable to the surgeon concerned, has since been given to the world by Mr. Wickham, of the Winchester Hospital;* and it is not impossible that something of the same kind may have existed in a case of my own: for I mentioned to you that it had happened to me, as well as to some more eminent surgeons, to have once cut into a patient's bladder without

* London Medical and Physical Journal, February 1829, p. 130.—Ed.

finding a stone. In the case to which I allude, the young gentleman rapidly recovered, and the mystery remains undeveloped; but into the particulars of that case I do not propose to enter: for, in my opinion, a surgeon never appears to less advantage than when, after a disappointment of this kind, instead of frankly acknowledging his error or regretting his misfortune, he enters into a tedious and hypothetical explanation, which seldom satisfies any one but himself.

Amongst other cases of disease of the urinary organs, the following very remarkable one presented itself on the 31st of October, in Walter Hay, æt. forty-five. "About two months ago was cut in the hospital for fistula ani, and discharged almost well. He now complains of a difficulty in passing his water, the half of which he states to be discharged by the anus. A large catheter may be easily passed into the bladder; the right lobe of the prostate is found to be enlarged and hardened, and is very painful when pressed between the catheter and the finger in the rectum."

After a few days' respite, during which the fact of his passing large quantities of urine by the rectum was fully ascertained, the case was accurately examined, and "the orifice of the sinus between the urethra and rectum was distinctly seen by means of the speculum ani: a probe was readily passed through it, and made to grate upon a catheter previously passed into the bladder."

On the following day, the 10th of November, the patient was brought into the theatre, and a full-sized catheter attempted to be passed, but, from the swelling and tenderness in the urethra, consequent upon the examination of the preceding day, it met with obstruction in the perineum, and could not be made to enter the bladder. Knowing, however, that the patient evacuated a great proportion of his urine freely by the natural passage, I was induced to persevere in my purpose of cauterizing the callous edges of the fistula, which was done with a redhot iron, the rectum having been previously dilated with the speculum.

"On the 11th, "more water" was reported to be "passing by the urethra, but he complains much of pain from pressure on the back part of the urethra, and about the anus." Fourteen leeches were applied, with relief; and on the 13th the following report was entered:

"Last night, as the pain and irritability of the urethra had very much abated, an elastic catheter, No. 12, was secured in the bladder," and he took an anodyne draught, with eighty drops of laudanum, at bedtime.

On the 14th, he was reported to have "passed a tolerable night, and experiences less pain from the catheter; pulse natural, tongue slightly furred; bowels relieved once; no water appears to be discharged by the anus." After this period the whole of his urine was expelled through the catheter, which lay in the bladder for about a fortnight or upwards: at the end of this time it was withdrawn; a puriform discharge, occasioned apparently by its presence in the urethra, gradually subsided, and on the 6th of December he was dismissed cured.

This belonged to a class of cases of all others the most troublesome in their treatment, and but too often unsatisfactory in their results. The cure from a single application of the cautery was more than I anticipated, and was no doubt greatly facilitated by the free and pervious state of the urethra, and by our being enabled to introduce a full-sized catheter into the bladder soon after the application of the cauterizing iron, before the eschar occasioned by it was detached. But, while this open state of the natural passage obviously contributed much to the cure, it presents to me a very considerable difficulty in accounting for the formation of this fistulous opening, which was more than an inch within the extremity of the gut.

Amongst the diseases of the vascular system, you have, during the present course, had an opportunity of seeing, under my care, two cases of temporal aneurism from arteriotomy, one of these occurred in a patient under treatment for another complaint; was of a very small size, and was cured by compression. The other that of Bernard M'Kenzie, æt. twenty-eight, is thus noticed in the journal: "Admitted on the 8th of December with a small aneurism of the anterior branch of the temporal artery. The swelling is about the size of a nut, of a livid colour, and pulsates very distinctly. Disease is the consequence of the bandage having been too soon removed after arteriotomy, practised about three weeks ago."

After having tried the effect of a hard compress over the tumor for several days without any obvious benefit, I was induced to remove the whole of it by an elliptical incision, including a small portion of the surrounding integuments. The inferior or proximal extremity of the artery bled freely, and was secured by a ligature, and the surface of the wound covered with a dossil of lint. On removing this some days afterwards, the sore appeared covered with healthy granulations, and the healing process went on progressively until the 2d of January, when he was dismissed cured.

The aneurism in this case appears, from the preparation which I now show you, to have been of the kind termed false, originating from a wound in the coats of an artery. You may here see a bristle passed through the caliber of the vessel, and a coagulum of blood lying contiguous to it, in a cyst formed of condensed cellular membrane.

This is, so far as my observation goes, the common description of aneurism arising from bleeding in the temple, nor have I indeed seen any other from this cause; but it is stated that this aneurism sometimes assumes a cellular form, and puts on all the characters of aneurism by anastomosis, a disease which is always most successfully treated by the excision of the whole tumor, where it is limited in extent and distinctly circumscribed.

In a case, however, like M'Kenzie's, I am disposed to think that the simple division of the arterial branch above and below the tumor, with the subsequent application of a compress, would prove not only an equally efficient, but also a speedier means of cure.

The frequent occurrence of these swellings, and the trouble and danger which occasionally arise from them, ought to render you careful in the adjustment of the compress after arteriotomy; an operation now become so common, that it is thought altogether below the notice of an experienced surgeon, and is often left to young men quite unacquainted with its vexatious results.

As a sequel to these cases, I would next mention that of James Paulin, æt. forty, who was admitted on the 16th of January. This man had come in from Berwickshire, greatly dissatisfied with the treatment of some of his medical advisers, and anxious to submit to any operation which might be deemed necessary for the cure of some "varicose veins in his right leg, which he first observed about six months ago, and which he attributed to constant riding on horseback. The saphena vein below the knee became first affected, and he has occasionally pain in the course of that vessel, and in the groin. There is much swelling of the veins below the knee, and considerable œdema of the leg. General health good; bowels slow."

Having given him a few days' rest after his journey, and administered some laxative medicine, I determined upon making trial of the mode of cure by caustic, lately recommended to the notice of the profession by Mr. Mayo, of the Middlesex Hospital; not, however, I own, without many doubts of its success; for, as I explained to you in lecturing upon this case, I was afraid that the caustic, like some other practices, would either do too little or too much; that it would either fail of the desired effect, or excite an active inflammation within the vein, the danger of which you have all been made acquainted with.*

On the 20th of January, a portion of caustic potass was made into a paste with soft soap, and applied over the course of the vein a little above the knee, its action being circumscribed by a piece of adhesive plaster previously fixed upon the thigh, with an oval aperture in it for the admission of the caustic. After having been allowed to remain for about seven hours, it was removed, and an emollient poultice applied; the eschar separated in about ten or fourteen days afterwards, and left the trunk of the vein exposed in the bottom of the wound to the extent of upwards of an inch; it was also to be felt hard and swollen for about an inch below, and about two or three inches above, the site of the eschar. The portion of it laid bare was expected to slough, it being seen black and apparently dead in the bottom of the sore: this, however, did not take place; the granulations, which were florid and healthy, gradually encroached upon it, and covered it over. The man now became impatient to leave the hospital, and to return to his home, expressing much gratitude for what had been done, and being satisfied that he was to obtain an effectual cure. He was with difficulty detained until the 13th instant, at which time the sore was cicatrising rapidly; and, what was remarkable, although the principal dilatation of the vein immediately below the knee did not appear to be much diminished while the limb was at rest, yet no sooner was it put in motion than the blood began to circulate freely through the collateral veins, and the swelling to disappear.

I am now, gentlemen, according to the established routine of attendance in this house, about to relinquish a duty, than which I have never discharged any other with more gratification to myself. The practical mode of instruction which best befits this chair, is one the most consonant to my taste and to all the habits of my life. Could I presume to think that our connexion as teacher and student had been as in-

* We have recently witnessed the treatment of several cases at the Middlesex Hospital, under Mr. Mayo's direction, by the method here referred to. In no instance that we have seen has inflammation of the vein, or any other bad consequence, ensued; while the cavity of the vein has been uniformly obliterated at the part where the caustic has been applied. By what process this takes place is obscure. Mr. Mayo applies the caustic paste at several points upon the varicose vein or veins, so as entirely to shut them out of the circulation.—Editors.

structive to you as it has been agreeable to me, I should then retire with the conviction of having accomplished a great public good, and of having contributed largely to the education of young men, who require only to have their studies well directed, to ensure their becoming useful members of their profession and blessings to society. But, gentlemen, when I look to the very extended experience and deep research which the surgical pupils of this hospital have long been accustomed to witness in the professor of clinical surgery, I cannot but fear that I may have come far short of the mark: you will, however, permit me to say that, in so far as my experience could be brought to bear on the cases under consideration, it has been fairly laid before you. But, while I have ever been most anxious to discharge my duty to the patients in this house, and to the students of this class, I have never made any display of a questionable zeal, nor have I any affected enthusiasm to support. Why, then, gentlemen, should I hesitate to avow that I now look forward with pleasure to the appointment of consulting surgeon, as one demanding less continued attention, and as one fairly earned by three-and-twenty years' employment in the responsible, and often laborious, duties of hospitals? I have no right, however, to promise myself any thing like a respite from my labours as a teacher. I must not forget that there is another body of young men, the students of military surgery, who have every claim to my best exertions, and to whose behoof I am bound to turn the extensive field of observation which still lies open to me as a consulting surgeon of the house. As a consulting surgeon, I shall still enjoy many valuable opportunities of adding to my own stock of experience, and of contributing to their information; as a consulting surgeon of this house, I shall ever retain a warm interest in the treatment of its patients, as well as in the instruction of its pupils; and, whenever you or your successors shall give me an opportunity of showing such interest, you will not, I trust, find me forgetful of the kind and respectful attention with which I have uniformly been honoured by the students of clinical surgery.

---

